# Supplementary material for: Causal Relationship between Aging and Anorexia Nervosa: A White-Matter-Microstructure-Mediated Mendelian Randomization Analysis
Source: Biomedicines. 2024 Aug 16;12(8):1874. doi: 10.3390/biomedicines12081874 (PMC11351342; doi:10.3390/biomedicines12081874)
Supplement: Supplementary file 1 [file biomedicines-12-01874-s001.zip › Supplementary Figure/Figure S4/Singe SNP plot of MR analysis for TL to ubm-b-1455.pdf]

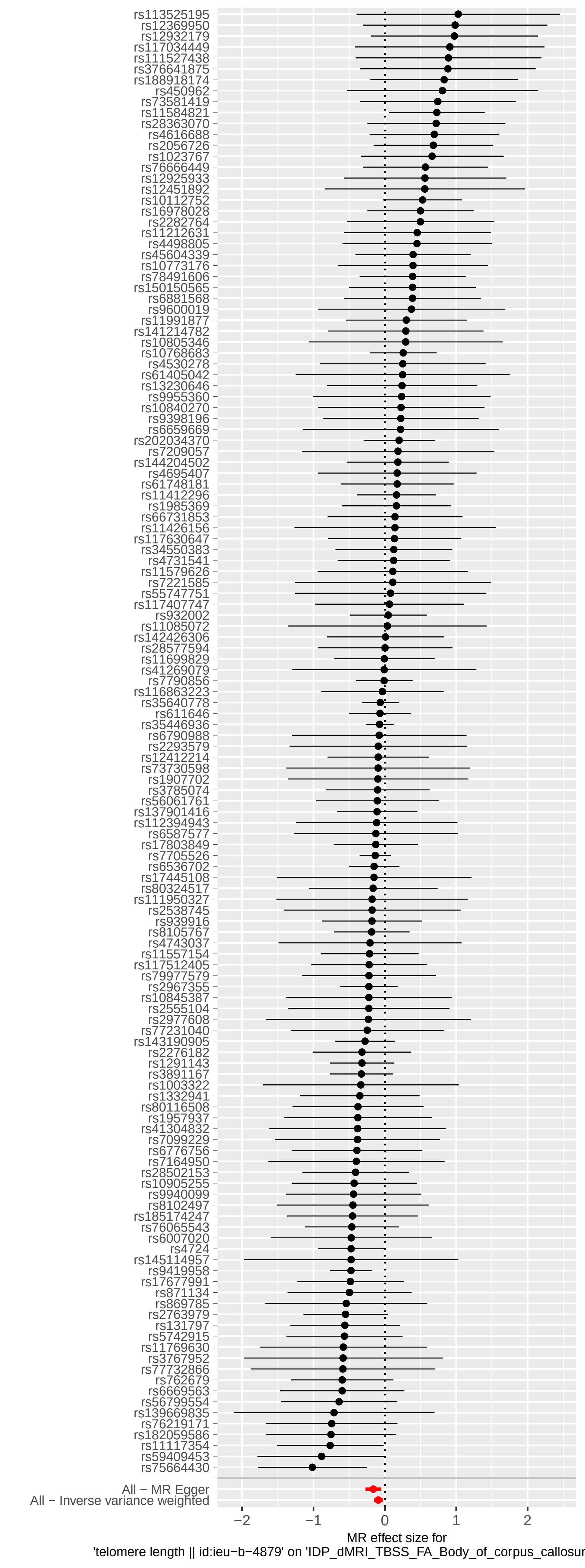
rs113525195
rs12369950
rs12932179
rs117034449
rs111527438
rs376641875
rs188918174
rs450962
rs73581419
rs11584821
rs28363070
rs4616688
rs2056726
rs1023767
rs76666449
rs12925933
rs12451892
rs10112752
rs16978028
rs2282764
rs11212631
rs4498805
rs45604339
rs10773176
rs78491606
rs150150565
rs6881568
rs9600019
rs11991877
rs141214782
rs10805346
rs10768683
rs4530278
rs61405042
rs13230646
rs9955360
rs10840270
rs9398196
rs6659669
rs202034370
rs7209057
rs144204502
rs4695407
rs61748181
rs11412296
rs1985369
rs66731853
rs11426156
rs117630647
rs34550383
rs4731541
rs11579626
rs7221585
rs55747751
rs117407747
rs932002
rs11085072
rs142426306
rs28577594
rs11699829
rs41269079
rs7790856
rs116863223
rs35640778
rs611646
rs35446936
rs6790988
rs2293579
rs12412214
rs73730598
rs1907702
rs3785074
rs56061761
rs137901416
rs112394943
rs6587577
rs17803849
rs7705526
rs6536702
rs17445108
rs80324517
rs111950327
rs2538745
rs939916
rs8105767
rs4743037
rs11557154
rs117512405
rs79977579
rs2967355
rs10845387
rs2555104
rs2977608
rs77231040
rs143190905
rs2276182
rs1291143
rs3891167
rs1003322
rs1332941
rs80116508
rs1957937
rs41304832
rs7099229
rs6776756
rs7164950
rs28502153
rs10905255
rs9940099
rs8102497
rs185174247
rs76065543
rs6007020
rs4724
rs145114957
rs9419958
rs17677991
rs871134
rs869785
rs2763979
rs131797
rs5742915
rs11769630
rs3767952
rs77732866
rs762679
rs6669563
rs56799554
rs139669835
rs76219171
rs182059586
rs11117354
rs59409453
rs75664430
All − MR Egger
All − Inverse variance weighted
−2
−1
0
1
2
MR effect size for
'telomere length || id:ieu−b−4879' on 'IDP_dMRI_TBSS_FA_Body_of_corpus_callosur
